# [Rectacted] *miR‑106b‑5p* promotes cell cycle progression of malignant melanoma by targeting *PTEN*

**DOI:** 10.3892/or.2023.8669

**Published:** 2023-11-20

**Authors:** Xu-E Chen, Pu Chen, Shan-Shan Chen, Ting Ma, Guang Shi, Ya Zhou, Ji Li, Liang Sheng

Oncol Rep 39: 331–337, 2018; DOI: 10.3892/or.2017.6099

Following the publication of this paper, it was drawn to the Editor's attention by a concerned reader that the colony formation data shown in Fig. 2C on p. 333 had already appeared in previously published articles written by different authors at different research institutes. Owing to the fact that the contentious data in the above article had already been published prior to its submission to *Oncology Reports*, the Editor has decided that this paper should be retracted from the Journal. The authors were asked for an explanation to account for these concerns, but the Editorial Office did not receive a reply. The Editor apologizes to the readership for any inconvenience caused.

